# Assessing the Outcomes of an Animal-Assisted Intervention in a Paediatric Day Hospital: Perceptions of Children and Parents

**DOI:** 10.3390/ani10101788

**Published:** 2020-10-01

**Authors:** Adriana Ávila-Álvarez, Jerónimo Pardo-Vázquez, Iván De-Rosende-Celeiro, Rita Jácome-Feijoo, Gabriel Torres-Tobío

**Affiliations:** 1 Occupational Therapy Research Unit in Non-Pharmacological Interventions, University of A Coruña, 15071 A Coruña, Spain; adriana.avila.alvarez@udc.es; 2 Paediatrics Service, A Coruña University Hospital Complex, 15006 A Coruña, Spain; jeronimo.jose.pardo.vazquez@sergas.es (J.P.-V.); rita.jacome.feijoo@sergas.es (R.J.-F.); 3 Department of Physical and Sports Education, University of A Coruña, 15071 A Coruña, Spain; gabriel.torres@udc.es

**Keywords:** animal-assisted intervention, day hospital, dogs, emotional well-being, occupational therapy, outcomes, paediatric illness

## Abstract

**Simple Summary:**

Scientific evidence of the effects of intervention with dogs as a non-pharmacological distraction strategy in child hospital care is still small. In addition, the results of implementation of animal-assisted programmes at a paediatric day hospital are not known. Thus, in this pilot study, we conducted preliminary research on the efficacy of an intervention based on the use of therapy dogs at a Spanish day hospital. An intra-subject quasi-experimental longitudinal design was used. The study showed that implementation of an animal-assisted programme at a paediatric day hospital is feasible. Children and parents both gave this programme their highest satisfaction rating. The results portray a significant improvement in the child’s self-assessment of their emotional well-being. In parallel, the parents confirmed this significant improvement in the child’s mood. The research suggests that conducting an animal-assisted session just before an outpatient medical procedure is an effective approach in the promotion of emotional welfare, a compromised area for a high percentage of paediatric patients during hospitalisation periods.

**Abstract:**

There is a growing interest in the use of animal-assisted intervention (AAI) as a non-pharmacological strategy to improve child welfare in hospitals. However, the efficacy of implementation of programmes based on activities with dogs in a paediatric day hospital is not known. An intra-subject quasi-experimental longitudinal design was used for the purpose of assessing the feasibility of such an intervention, as well as exploring the perceptions of its outcomes by children and parents/guardians. A total of 55 children in outpatient care at a paediatric day hospital participated in an AAI session. The application of this intervention was feasible. Self-assessment by the paediatric patients revealed a statistically significant improvement in their emotional state after the session, which was large in size. Parents confirmed this improvement in their child, perceiving significant changes in their mood, which were large in size. This study suggests that AAI is an effective approach when it comes to promoting the emotional welfare of children during their stay in hospital care environments. Participating in recreational occupations with dogs could contribute in a relevant and efficient way to the development of significant and gratifying experiences and to a more positive perception of healthcare centres on the part of children.

## 1. Introduction

Hospital stays can compromise the emotional welfare of children. A relevant percentage of children evidence psychological distress during their hospitalisation, stemming from a complex combination of negative emotions such as anxiety, sadness or fear [1,2,3,4,5]. These experiences are also common if they are outpatients at the hospital [6,7]. The main concerns for the paediatric population regarding hospital stays are invasive and painful procedures, separation from their families and social environments, remaining in an unfamiliar environment and the loss of self-determination [3,8]. Despite the importance of reducing the emotional suffering of children in medical care, the development of non-pharmacological strategies to deal with this problem has been limited [9,10]. The use of distraction is common [11]. This is a simple and economical technique based on the modification of the environment, consisting in shifting the child’s focus from distress-triggering negative stimuli related to the illness and the medical procedures to non-medical elements that are more attractive and pleasant [12,13,14]. In 2017, a meta-analysis of the impact of distraction in children with an oncological disease concluded that it is effective in the reduction of pain in healthcare environments [15]. Regarding medical procedures that use needles, a recent systematic review supported the incorporation of distraction strategies with the aim of reducing pain and psychological distress among children, although the quality of the evidence was low, arguing for the need to conduct more research in this field [11]. 

There is a growing interest in the use of animal-assisted intervention (AAI) as a distraction-based coping strategy for paediatric patients [16,17,18,19]. This is a complementary treatment method designed to improve well-being and health, characterised by interaction with specifically trained animals during planned activities led by a specialised professional team [20,21]. The International Association of Human–Animal Interaction Organizations (IAHAIO) [22] defines AAI as an “intervention that intentionally includes or incorporates animals in health, education and human service for the purpose of therapeutic gains in humans”. In children’s hospitals, health professionals such as occupational therapists have conducted various AAI experiences with dogs. The positive friendships and affection resulting from the interaction between the child, the dog and the therapist are the basis for this type of intervention and may act as a key distraction factor [17,23].

In recent years, several systematic reviews have examined empirical research on the efficacy of AAI in healthcare for paediatric patients. A 2016 review looked at a heterogeneous assortment of 36 studies on the efficacy of AAI in healthcare centres, of which only 22.2% included samples of hospitalised children [24]. It was observed that the development of simple hygiene protocols was effective in the minimisation of risks associated with the use of dogs in hospitals, such as infections or allergies. This review shed light on improvements in pain, stress and anxiety levels. However, it concluded that findings must be interpreted cautiously due to the insufficient number of works analysed, the use of reduced sample sizes and the general low quality of literature on this subject. Similarly, a 2017 review of interventions with dogs in health centres that mainly included research focused on adults concluded that this strategy has positive effects, especially in people with psychiatric disorders, although these are small or moderate in size [25]. Seven studies (38.9%) assessed interventions with paediatric patients: four of them with children who visited the centre as outpatients for a physical examination or dental procedure; the rest with children or adolescents with mental illnesses. Although emotional benefits were shown, the authors highlighted that the evidence was weak, partly due to the inclusion of research with a limited sample size. The most recent literature on the efficacy of programmes with therapy dogs in paediatric hospitals offers contradictory results. In a 2017 clinical trial with hospitalised children, the participants in the intervention with dogs did not experience a significantly higher improvement in their emotional well-being compared to the children in the control group [26]. Among children with an oncological condition, two before–after design studies supported the benefits of this type of AAI regarding the self-assessment of their emotions [17,27]; however, a 2018 randomised controlled trial did not find significant improvements in children’s perception of their degree of anxiety or quality of life [21].

Scientific evidence of the effects of intervention with dogs as a non-pharmacological distraction strategy in child hospital care is still small. Reduced sample size is one of the key limitations, and few studies have included younger paediatric patients, that is, those at the pre-school stage. Furthermore, in paediatric hospitals, the previous literature has explored the impact of dog-assisted interventions in a variety of inpatient settings, including acute care settings, oncology wards and psychiatry units, ranging from simple pet visits in the patient’s room to the implementation of various sessions over several weeks [24,25]. However, the results of implementation of AAI programmes at a paediatric day hospital are not known. The main differentiating feature of this kind of medical–surgical unit is the short length of stay (less than 14 h). It provides general paediatric patients with a child-friendly environment for comprehensive outpatient care with a diagnostic, therapeutic and/or surgical purpose. Thus, in this pilot study, we conducted preliminary research on the efficacy of an intervention based on the use of therapy dogs at a Spanish day hospital. Based on the research literature, it was hypothesized that AAI improves the emotional well-being of the children admitted to the day hospital. The specific aims were to examine the feasibility of this intervention as well as to explore the perceptions of its outcomes by the participating children and their parents/guardians in relation to the emotional well-being of the paediatric patient and satisfaction with the programme. 

## 2. Materials and Methods 

### 2.1. Ethical Statement

The study protocol was approved by the regional ethics committee (Research Ethics Committee of Coruña-Ferrol, identification code 2017/178). Each child’s parents or legal guardians received full verbal and written information about the research. It was explained to them that participation was voluntary. Subsequently, they signed an informed consent form to authorise the participation of their child in the research. In addition, only those children who, alongside their parents/guardians, showed themselves willing to participate in the AAI were recruited for the research. Confidentiality was preserved in accordance with the European Union General Data Protection Regulation 2016/679. The study followed the tenets of the Declaration of Helsinki [28]. 

### 2.2. Study Design

An intra-subject quasi-experimental longitudinal design was used for the purpose of examining the effects of participating in an AAI session conducted at a paediatric hospital unit.

### 2.3. Participants

The following were the inclusion criteria for the study: (a) children older than 2 and younger than 16 years of age; (b) having enough mental, physical and communication abilities to understand and follow the assessment procedures and the AAI session in accordance with the assessment of the unit’s specialist doctor; and (c) no known history of allergy to or phobia of dogs. We did not include children with infectious diseases, isolation precautions, asthma, respiratory disease with an obstructive component or in an immunosuppressive state, nor those who showed excessive fear, anxiety or discomfort in the presence of a dog.

### 2.4. Animals Involved

Three medium-sized dogs were used (two females and a male). Their average age was 4.3 years. Two were Labrador Retrievers and one was a Golden Retriever. The animals completed a specific training process as therapy dogs to develop a calm, docile and obedient temperament and to be friendly to children and be able to easily adapt to different situations. The dogs were trained and brought into the day hospital by registered dog trainers from a specialised external centre (Montegatto). The IAHAIO standards were met [22]. All animals were carefully examined periodically by an authorised veterinarian, ensuring that their health was good and that they complied with basic standards of vaccination, anti-parasitic treatment and hygiene. The health protocol specifically designed for this study by the hospital’s Preventive Medicine Service was followed. This protocol establishes the procedure for the access and exit of dogs in order to restrict their circulation and minimise contact with patients and visitors, as well as hygiene measures for hand washing, changing of clothes and exhaustive disinfection/cleaning of surfaces and equipment. Furthermore, the research protocol set obligations for compliance with strict indications related to the health, hygiene and washing of the animal. 

### 2.5. Setting

The research was carried out at the Paediatric Day Hospital of the A Coruña University Hospital Complex in the Spanish city of A Coruña. This hospital unit belongs to the public healthcare system and serves a diverse population of patients under 16 years of age who live in urban and rural environments. It provides comprehensive outpatient care from 08:00 to 22:00 h, Monday to Friday, through the coordinated work of specialist doctors from various hospital departments and nursing professionals. It has 8 beds, 12 paediatric chairs and a procedure room. Most patients come to the day hospital from home (others are already hospitalised). Diagnostic, therapeutic and surgical procedures are conducted, such as pre- and post-operative care of surgery outpatients; administration of chemotherapy cycles, intrathecal therapy, antibiotics and corticosteroids; blood product transfusion; immunoglobulin treatment; sedation for diagnostic and therapeutic procedures; and blood gas tests, cultures, lumbar punctures and bone marrow biopsy or aspiration. At the end of their stay at the day hospital, they may return home or receive care in one of the centre’s wards. 

The research was conducted for a period of six consecutive months, one day per week (Wednesday). The participants were patients at the Paediatric Day Hospital during the studied period. The specialist doctor in charge of the unit recruited them. Each day he invited three patients to participate. These were selected randomly from the electronic register of patients who were receiving care at the unit on that day. If the child did not meet the eligibility criteria or their parents/legal guardians did not authorise their participation in the study, the doctor continued with the selection process until recruiting a maximum of three children per day. Demographic information and clinical data related to admission to the day hospital were collected. The presence of dogs in participants’ homes was examined (dog ownership vs. no). The current ownership of cats was also examined (cat ownership vs. no).

### 2.6. Intervention

The intervention consisted of a single AAI session that lasted for approximately 20–30 min. This duration was consistent with prior experience of the research team in the implementation of AAI programmes at this hospital [29]. The session was held before undergoing the medical procedure. AAI is a complementary strategy designed to provide a significant recreational distraction serving as a diversion during a hospital stay as well as to promote the well-being of the children admitted to the day hospital. It uses the relationship and activities with a trained dog as an intervention method, with the mediation of a specialised therapist. 

With the aim of promoting animal welfare, the sessions were individual in nature, with the presence of the participant, a therapy dog, a therapist and at least one of the father, mother or legal guardian. The sessions took place in a private area of the day hospital, with natural light coming in from several windows, between 10:00 and 12:30. They were planned and conducted by an occupational therapist who had specialised training and extensive experience in AAI. The therapist acted as a mediator and facilitator in the interaction between the participant and the dog, encouraging the active participation of the child, and their parents through the individualised use of the following behavioural strategies: positive reinforcement, modelling and feedback. 

A semi-standardised approach was taken, characterised by using the preferences and choices of each participant as a starting point. A key principle was the involvement of the participant in the selection of activities in order to promote their motivation and interest. The therapist began the session with a brief conversation with the child/adolescent about their experiences with dogs (name of their dogs, breed, age, favourite games with their pet, how they care for their pet, etc.). Next the repertoire of activities that could be done was presented, encouraging the participant to choose the activities they would take part in. Each participant could choose how to structure their time for the duration of the session. In addition, instances of free and spontaneous play with the dog were permitted, as well as periods of rest in the company of the animal. 

Various types of activities were used, classified into five major groups. The first group comprised “activities involving getting to know the animal”, consisting in identifying its breed and finding out its name, asking the therapist questions about the dog, exploring its physical characteristics and recognising its response to basic human actions. The second group consisted of “activities involving interaction with the dog”, such as speaking to the animal and communicating thoughts or feelings to it, establishing physical contact through touch, embracing it, sitting close to it, cuddling up with it, interacting freely with it, engaging in basic obedience activities (“sit, down, shake”, etc.) and rewarding it. The third group included “activities involving caring for the animal”: preparing its water and food, giving it water and food (a treat), learning basic health and hygiene habits such as brushing and mouth and nail care procedures, learning methods for holding the dog, taking it for a walk or understanding its needs regarding rest. The fourth group consisted of “distractions involving playing with the dog”, such as recreational activities based on throwing a ball for the animal to pick up and bring back, games involving finding objects, games involving obstacles, passing through a tunnel of colours or jumping through a hoop, singing songs, etc. Finally, a fifth group of activities was added for the adolescent population relating to “features of intervention with therapy dogs”, which consisted of explaining to the participants what a therapy dog is, what its qualities are, how is it trained and why its use in hospitals is beneficial. The inclusion of this last group of activities stemmed from prior experience of the research team with adolescents, and it attempted to adjust the AAI to the motivations and interests that characterise this stage of life. 

### 2.7. Outcome Measures and Data Collection

Outcome measurements were classified into the following domains: emotional well-being of the child during their stay at the day hospital and satisfaction with the AAI. Kahneman et al. [30] describe emotional well-being as the emotional quality of an individual’s experiences, i.e., the intensity of experiences of joy, anxiety, sadness, anger and affection “that make one’s life pleasant or unpleasant”. In accordance with pre-existing literature, the outcomes in the emotional domain refer to the efficacy of the intervention upon the child’s mood or changes in the perception of feelings such as anxiety, fear or sadness [31].

Data was collected through an evaluation conducted immediately before (baseline assessment or T1) and after (second assessment or T2) the AAI session using questions for the participant and for one of their parents/legal guardians, posed by a member of the research team outside the room used for the session. The research team members were not part of the professionals who provided medical care for the patient at the unit. Before the session, the parents/guardians answered a questionnaire regarding their knowledge of this type of intervention and their previous experience participating in an AAI session.

#### 2.7.1. Perceptions of the Children

The Facial Image Scale (FIS) standardised tool [32,33] was the primary outcome. The FIS instrument was used to know the child’s self-assessment of their emotional state before and after the AAI session. This consists of five face drawings that range from a very happy face on one end to a very sad face on the other end. The child is asked to select the face that best represents how they feel at the time, and a score from one to five is obtained: the face with the most positive expression is worth one point; the face with the most negative expression, five points. This scale is a valid tool for children aged three or over in a clinical context; thus, it was used with participants who were at least three years of age. Two studies supported the employment of FIS as a tool that is easy to use, requires little training and can be quickly applied (in less than one minute) [32,33]. In a sample of 100 participants between 3 and 18 years of age, there is a strong correlation between FIS scores and those obtained in the Venham Picture Test [34], a valid picture scale to measure the “state” anxiety of small children (*r* = 0.7) [32]. Hence its concurrent validity is good. In addition, reasonably good agreement was observed between the parent’s evaluation of their child’s state and the score given by the child through the use of FIS (*k* = 0.66) [33].

In order to determine the degree of satisfaction with the AAI, the question “Did you like the activity (done with the dog)?” was posed to the participant child immediately after the end of the session. This question comes from one of the items of the Paediatric Interest Profiles (PIP) standardised tool [35]. For each of the recreational activities in which the child takes part in their day-to-day life, this tool asks the child whether they like said activity and presents them with a sheet with three mutually exclusive answer choices: “a lot” (3 points), accompanied by a picture of three stars; “a little” (2 points), accompanied by two stars; and “not at all” (1 point), accompanied by one star. The participant answered the question by colouring in or circling one of the answers, reflecting how much they enjoyed the session. If the child could not read, the instructions were read to them. Since this tool is not recommended for use with children under five, in this study the question was posed only to participants who were at least five years of age.

#### 2.7.2. Perceptions of the Parents/Guardians

The scale developed in the Kaminski et al. [36] study was used before and after the AAI session in order to know how the parents/guardians rated the child’s emotional well-being during their time at the day hospital. This tool was designed to determine the impact of the use of therapy dogs on a sample of hospitalised children. It includes 4 items: happy, lonely, scared and relaxed. One of the parents or guardians is asked to rate their perception of the child’s current mood. Each item is rated on a five-point scale, from 1 to 5. A score of 5 represents the most positive mood. A total score is obtained by adding all the items, with a possible range from 4 to 20 points. Cronbach’s alpha coefficients above the 0.7 value recommended by pre-existing literature [37] were found when the internal consistency of this scale was evaluated on a sample of hospitalised paediatric patients; hence, its reliability was good. 

Additionally, after completing the AAI session, one of the parents/legal guardians rated their degree of satisfaction with the intervention on a scale from 0 (not at all satisfied) to 10 points (very much satisfied).

### 2.8. Data Analysis

Statistical analyses were performed using the IBM SPSS 22.0 (IBM Corporation, Armonk, NY, USA). Descriptive statistics were used to summarise the results. The categorical variables were reported as frequencies and percentages. The Kolmogorov–Smirnov test was used to determine the normal distribution. The variables that followed a normal distribution were described using the mean and the standard deviation (SD); those that did not follow the normal distribution and the ordinal variables, through the median and the first and third quartiles (Q1–Q3). 

The relationship between a selection of descriptive variables (age, gender, dog ownership, hospitalised before admission to the day hospital and type of medical procedure) and the primary outcome in the T1 and T2 evaluations was examined. The Spearman correlation coefficient was used to evaluate the association between age and the primary outcome. The Mann–Whitney *U*-test was used to determine the relationship between the dichotomous variables and the primary outcome. The Kruskal–Wallis test was used to explore the association between the type of medical procedure and the primary outcome; if *p* was significant, a Bonferroni test was performed as a post hoc test.

Regarding the FIS and the scale of Kaminski et al., 2002, the changes in the scores between the T1 and T2 assessments were tested for significance by means of the Wilcoxon´s signed-rank nonparametric test. The effect size (*r*) (ES(*r*)) was calculated by dividing the *Z* statistic of the Wilcoxon´s tests by the square root of the total number of observations. An ES(*r*) of 0.10 constitutes a small effect, 0.30 a medium effect and 0.50 a large effect [38]. The level of significance was set a priori at *p* < 0.05 (two-sided). 

## 3. Results

During the period studied, the doctor in charge of the unit invited a total of 64 patients admitted to the day hospital to participate; six children (9.4%) were not included for the following reasons: no consent was given by parents/guardians or the child did not wish to participate (*n* = 4); known dog allergy (*n* = 1); and excessive fear, anxiety or discomfort in the presence of a dog (*n* = 1). Of the 58 patients who met the study criteria and agreed to their participation, 3 children (5.2%) were unable to take part in the AAI session due to timetable clashes with the medical care that they were receiving at the unit. Thus, the final sample consisted of 55 children. All participants completed the AAI session and the data collection process.

Table 1 details the information gathered in the initial assessment for the purpose of describing the characteristics of the sample. The median age was 8 years. Among those who took part, 20% were adolescents, being at least 13 years of age, in accordance with the definition of this stage of life in the pre-existing literature [31]. There was a slightly higher percentage of female participants (50.9%). There were no significant differences between girls and boys with regard to age (*p* > 0.05). More than half the sample (52.7%) had a dog in their home or had had one in the past, making up the group we called “dog ownership”. At the baseline, the number of participants who had a cat at home was smaller (27.3%); 78% of the sample came to the day hospital from home as outpatients. Regarding the health condition that motivated their admission to the unit, general paediatric surgery (36.3%) and endocrinology (18.2%) groups were the most notable. Most participants were admitted to the day hospital to undergo a diagnostic (49.1%) or surgical (41.8%) procedure.

### 3.1. AAI: Prior Experiences and Progression of the Sessions

The fraction of parents/guardians who already knew about AAI was under a quarter of the sample (23.6%). Only one participant had prior experience participating in an AAI session (Table 2).

The mean duration of the AAI session was 25.2 min (SD 6.3). The majority of relatives who accompanied the child during the session was their mothers (Table 2). Once the session began, no child evidenced a desire to terminate it early, nor were any withdrawn at the request of their parents/guardians. The therapist and the research team verified that no patient evidenced discomfort, anxiety or fear in the presence of the dog. No issues of allergic reaction to the dog were reported either. Finally, no events were recorded of aggressions towards the participants on the part of the dogs, damage to the room’s furniture, incidents involving breaking the safety health standards set or complaints from the public or the hospital staff.

### 3.2. Perceptions of the Children

#### 3.2.1. Results in the FIS Instrument

Among the participants who were at least three years old (*n* = 52), the median score in the initial evaluation with the FIS tool was 2 points. After the AAI session, the median score was 1 point, which corresponds to the choice of the face that shows the most positive emotion out of the possible five. As shown in Table 3, statistically significant changes were found when comparing the FIS scores obtained immediately before and after the session. These changes portray an improvement in the child’s self-assessment of their emotional well-being (*p* < 0.001). The effect size (ES (*r*)) was 0.50.

The FIS instrument was associated with the type of medical procedure in the T1 evaluation (*p* = 0.025). The FIS scores of the diagnostic procedure group were significantly lower than those in the surgical group (*p* = 0.024). The FIS scores were not significantly associated with other descriptive variables.

#### 3.2.2. Satisfaction with the AAI

Regarding the PIP question “Did you like the activity (done with the dog)?”, all participants who were at least five years old (*n* = 46) selected the response corresponding to the best possible evaluation of the AAI session, that is, the option “a lot” (Table 3).

### 3.3. Perceptions of the Parents/Guardians

#### 3.3.1. Outcomes with Respect to the Child’s Emotional Well-Being

Table 4 details the level of significance and the size of the effect of the changes identified in the evaluation conducted by the parents/guardians regarding the mood of the child on the Kaminski et al. [36] scale. Statistically significant changes were observed when comparing the total score on this scale before and after the AAI, reflecting a perception of a significantly better emotional state (*p* < 0.001). The size of this effect (ES (*r*)) was 0.56. There were significant improvements in the four items on the scale (*p* < 0.001). The largest effects were obtained for the items “happy” (ES (*r*) = 0.49) and “relaxed” (ES (*r*) = 0.48).

#### 3.3.2. Satisfaction with the AAI

On a scale from 0 to 10, the level of parent/guardian satisfaction with this intervention was the highest possible, the median being 10 points (Table 4).

## 4. Discussion

Providing scientific evidence of the feasibility of interventions with dogs at a paediatric day hospital through the responses and perceptions of children and parents was the main contribution of our research. In addition, this is the first study specifically designed to evaluate the outcomes of AAI in this type of outpatient unit. We recruited a sizeable sample in comparison with the prior existing literature for the purpose of overcoming one of the main limitations of works in this area [24,25]. Other strengths worthy of note owing to their infrequent nature in similar research were the examination of potential associations between the outcomes and the presence of dogs in homes, the enrichment of findings through the determination of effect sizes and the exploration of the perspectives of younger children, at the pre-school stage, through a standardised tool.

This research addressed the feasibility of an intervention based on the use of therapy dogs at a Spanish day hospital. Our results support the feasibility of and need for future large-scale studies on the effects of an AAI programme at a paediatric day hospital. We demonstrated that recruitment and data collection were feasible. Reasons such as the large sample size reached in a small-size hospital unit during a reduced period of time, as well as the high level of satisfaction of children and parents, were arguments for the acceptability of AAI. Consent on the part of the parents was predominant. Moreover, no complications that could endanger the health of the paediatric patients were observed. Children’s responses to the dogs were positive in all cases, completing the sessions without behaviours related to anxiety or fear of the animal or allergy problems. The safety precautions defined in the research protocol were complied with.

Another aim of this work was the evaluation of the outcomes. The findings suggest that participating in an AAI session was an effective approach towards improvement in emotional well-being. Among the participants, of children with acute or chronic medical conditions, their emotional state before the intervention was predominantly positive, which is consistent with previous studies that showed a lower frequency of emotional and behavioural issues in children who were admitted to hospitals as outpatients in comparison to those hospitalised for more than 24 h [39,40]. Although initial evaluations were relatively favourable, it is important to highlight that children and parents/guardians both perceived significant changes in emotional well-being after the AAI. Self-assessment by the paediatric patients revealed a large-sized affective improvement. Using a picture scale allowed us to know the feelings of the younger participants. Compared to verbal self-assessment methods, tools based on face drawings are easier to understand by younger children due to their lower level of development in the intellectual and language areas [32]. In parallel, the parents confirmed this improvement in the child’s mood, perceiving changes in the total score on the analysed scale. When evaluating the different types of emotions separately, gains were medium in size. After completing the session, the parents perceived that their children were significantly more cheerful and relaxed, as well as less fearful and socially withdrawn. The positive impact of AAI on the emotional well-being of the children during their time at the day hospital is consistent with the reduced scientific literature currently available on activities with dogs in healthcare environments, although it is necessary to emphasise the difficulty in comparing our findings with prior research due to the heterogeneous nature of study designs, settings, samples and methods for measuring outcomes. Most research conducted with hospitalised children evaluated a single AAI session, as did this study, but the average duration of our intervention was longer, since previous research included sessions that lasted between 10 and 20 min [18,19,26,27,41,42,43,44]. However, we believe that our intervention strategy could represent an efficient use of the limited resources available in healthcare, given the optimal results obtained with a single session lasting approximately 25 min, the small staff numbers involved and the reduced use of supporting materials.

This study supported the benefits of interactions between paediatric patients and dogs, confirming the findings of previous research work that proposed potential explanations for the positive impacts of this intervention. The “biophilia hypothesis” has suggested that human beings display an innate biological tendency to establish connections with other forms of natural life, especially with animals, visible from an early age [45,46]. One reason for this may be the existence of physical and behavioural features that are similar between humans and animals such as dogs [47]. Throughout history, dogs have been our main animal companions owing to their predisposition to observe human relationships and to display affective behaviours that are non-discriminating and free of prejudice [48,49]. Their unconditional acceptance of people favours the forging of friendship bonds and companionship, as well as the development of perceptions of safety, relaxation and comfort [16,17,23,44,50]. As an example of the pleasant effects of interaction with dogs, it has been observed that application of AAI in paediatric healthcare centres produces significant physiological improvements in indicators such as heart rate [51], arterial systolic blood pressure [42,51] or the level of cortisol (a hormone linked to stress) in blood plasma [16]. At the same time, the potential distracting effect produced by the interaction with dogs could explain its psychological benefits. Participating in recreational activities with these animals helps the child focus their attention on positive and gratifying stimuli, so they will pay less attention to more healthcare-related elements which they might associate with fear and pain, with positive repercussions on stress levels and emotional well-being [17,18,51]. In addition, some research in paediatric hospitals has revealed that AAI is more effective than other recreational distraction interventions [36,42]. Finally, another feature of AAI that may help the child’s better adaptation to a hospital setting is the perception of control over their environment. As highlighted by the Lazarus and Folkman (1984) model [52], cognitively recognising scenarios or environmental circumstances as threatening or dangerous is a key factor in the development of stress responses, and perceiving a lack of control over said threat represents a fundamental contribution to this cognitive assessment. In line with this theory, the impact of AAI is relevant because it allows the child to increase their degree of control over the unknown hospital world, since as their motivation and active involvement takes centre stage in the intervention, their choices and interests are prioritised and the performance of those activities that are most significant for each child is promoted [36]. Engaging in activities with the dog that are selected by the child, in a context of self-determination, peacefulness and enjoyment, contributes to improving the subjective perception of hospital settings by the paediatric population.

Some limitations should be mentioned. Firstly, it is important to highlight that there was no control group. The changes shown in the research suggest a positive effect of AAI due to the lack of similar non-pharmacological distraction strategies at the unit studied, the chosen method of collection of information where outcomes were measured immediately before/after the session, and the consensus observed among children and parents giving a maximum rating to describe their satisfaction. However, we cannot rule out the potential influence on the findings of other aspects related to the attention received during their stay at the hospital, such as their relationship with the healthcare team or the prescribed pharmacological treatment. The absence of information about potentially important variables, such as the degree of severity of medical diagnoses or comorbidity, was also a weakness. Furthermore, we recruited children who received care at a single hospital which serves a small geographical area, and these children were mostly admitted for diagnostic or surgical procedures. Receiving treatment was the reason for admission to the day hospital for only 9% of the subjects. Therefore, our outcomes may not be generalisable to other day hospital healthcare services. In addition, the voluntary nature of the assessed intervention may have implied the selection of a sub-group of patients in the unit, specifically, those with a greater predisposition towards interaction and play with animals. Lastly, it is not known whether the positive effects remained beyond the period studied, to what degree or for how long after the activity.

This study adds to the body of evidence for future research in this area. In order to confirm these preliminary outcomes, the next step must be to plan a randomised controlled trial. For the comparison group, a sample of patients belonging to the same population as the one studied but who receive the standard care at the unit could be recruited or, preferably, a sample participating in an active, recreational distraction activity that has been positively evaluated in previous literature and is different to the AAI. Although our sample size is large compared to similar studies on AAI in hospitalised children [17,18,21,26,27,41,42,43], the large-scale implementation of experimental designs, with a sample consisting of subjects from several paediatric day hospitals from across the country, would favour the generalisability of the findings and would provide stronger scientific evidence of the benefits of these programmes. Another recommendation for future research is the incorporation of qualitative methods, such as semi-structured interviews, in order to obtain a more detailed picture of the subjective views of participants in relation to their experiences of interaction with the dog, the benefits obtained and the aspects that should be improved in the intervention.

## 5. Conclusions

The outcomes reveal that the innovative implementation of an intervention based on activities with therapy dogs at a paediatric day hospital is feasible and appears to significantly improve the emotional state of the children. The implementation of this type of AAI seems to be feasible considering the level of participation of paediatric patients in this intervention, the high satisfaction expressed by patients and parents and the absence of adverse events. The research suggests that conducting an AAI session just before a medical procedure, especially a diagnostic or surgical one, is an effective approach in the promotion of emotional welfare. Participating in recreational activities with the animal favours children’s enjoyment and distraction during their stay at the hospital facility; thus, this type of non-pharmacological strategy could make a relevant and efficient contribution to the development of significant experiences and more positive perceptions of healthcare environments on the part of children.

## Figures and Tables

**Table 1:** Descriptive characteristics of the participants at baseline (*n* = 55).

Sample Characteristics	Value or *n* (%)
Gender Female	28 (50.9)
Age (years) Median (Q1–Q3) Range	8 (6–12) 2–15
Dog at home Currently Dog ownership: in the past or currently	26 (47.3) 29 (52.7)
Current cat ownership	15 (27.3)
Hospitalised before admission to the day hospital No	43 (78.2)
Health condition that led to admission to the day hospital General paediatric surgery Endocrinology Oncology General paediatrics Haematology Nephrology Other (e.g., cardiology, gastroenterology, infectology)	20 (36.3) 10 (18.2) 6 (10.9) 5 (9.1) 3 (5.5) 3 (5.5) 8 (14.5)
Type of medical procedure Diagnostic procedure Surgical procedure Treatment	27 (49.1) 23 (41.8) 5 (9.1)

Q1: first quartile; Q3: third quartile.

**Table 2:** Animal-assisted intervention (AAI): prior knowledge and characteristics of the session (*n* = 55).

Parents/Guardians: Knowledge at Baseline	*n* (%)
Knowledge of the AAI No	42 (76.4)
Have previous experience with AAI Yes	1 (1.8)
**AAI session**	**Value or *n* (%)**
Duration Mean (SD) Range	25.2 (6.3) 14–38
Parents/guardians participating in the session Only the mother Only the father Both Grandparents	28 (50.9) 11 (20) 16 (29.1) 6 (10.9)

AAI: animal-assisted intervention; SD: standard deviation.

**Table 3:** Outcome measures of the animal-assisted intervention (AAI): child/adolescent self-assessment.

Emotional State ^a^					
	T_1_	T_2_	*p*-value		Effect size
	Median (Q1–Q3)	Median (Q1–Q3)			
Facial Image Scale ^b^	2 (1.25–3)	1 (1–1)	*p* < 0.001 ^c^		0.50
**Satisfaction ^d^**					
Did you like the activity with the dog? ^e^ A lot A little Not at all				46 (100) 0 0	

AAI: animal-assisted intervention; T_1_: before the AAI session; T_2_: after the session; Q1: first quartile; Q3: third quartile; ^a^ Participants aged ≥ 3 years; *n* = 52; ^b^ Higher score indicates more negative affect; possible range = 1–5; ^c^ Indicates significant finding (*p* < 0.05); ^d^ Participants aged ≥ 5 years; *n* = 46; ^e^ Data are presented as *n* (%).

**Table 4:** Outcome measures of the animal-assisted intervention (AAI): perceptions of the parents/guardians (*n* = 55).

Emotional State of the Child/Adolescent ^a^					
	T_1_	T_2_	*p*-value		Effect size
	Median (Q1–Q3)	Median (Q1–Q3)			
Happy Lonely Scared Relaxed Total score	4 (3–5) 5 (4–5) 4 (3–5) 3 (3–5) 15 (13–18)	5 (5–5) 5 (5–5) 5 (5–5) 5 (4–5) 20 (18–20)	*p* < 0.001 ^b^ *p* < 0.001 ^b^ *p* < 0.001 ^b^ *p* < 0.001 ^b^ *p* < 0.001 ^b^		0.49 0.37 0.46 0.48 0.56
**Satisfaction ^c^**					
Satisfaction with the AAI Median (Q1–Q3) Range				10 (10–10) 6–10	

AAI: animal-assisted intervention; T_1_: before the AAI session; T_2_: after the session; Q1: first quartile; Q3: third quartile; ^a^ Based on the scale of Kaminski et al., 2002; higher score indicates more positive mood; possible range = 1–5; ^b^ Indicates significant finding (*p* < 0.05); ^c^ Possible range = 0–10.
